# Lead Exposure in Infancy and Subsequent Growth in Beninese Children

**DOI:** 10.3390/toxics10100595

**Published:** 2022-10-08

**Authors:** Shukrullah Ahmadi, Jérémie Botton, Roméo Zoumenou, Pierre Ayotte, Nadine Fievet, Achille Massougbodji, Maroufou Jules Alao, Michel Cot, Philippe Glorennec, Florence Bodeau-Livinec

**Affiliations:** 1 Centre of Research in Epidemiology and Statistics/CRESS, Université de Paris, INSERM, INRA, 75004 Paris, France; 2 EPI-PHARE, Epidemiology of Health Products, 93200 Saint-Denis, France; 3 Faculty of Pharmacy, University Paris-Sud, 92296 Châtenay-Malabry, France; 4 Institut de Recherche pour le Développement (IRD), Paris Descartes Université, 75004 Paris, France; 5 Département de médecine sociale et préventive, Université Laval and Centre de toxicologie du Québec, INSPQ, Québec, QC G1V 5B3, Canada; 6 Faculté des Sciences de la Santé, Cotonou 01 BP 526, Benin; 7 Paediatric Department, Mother and Child University and Hospital Center (CHU-MEL), Cotonou 01 BP 107, Benin; 8 Univ Rennes, EHESP, Inserm, Irset (Institut de Recherche en Santé, Environnement et Travail)—UMR_S 1085, 35000 Rennes, France

**Keywords:** heavy metals, lead exposure, growth outcomes, cohort study

## Abstract

Studies suggest that elevated postnatal blood lead levels (BLLs) are negatively associated with child growth. This study aimed to investigate the associations of childhood BLLs at age one year and growth outcomes at age six years (*n* = 661) in a cohort of children in Allada, Benin. The growth outcomes studied are weight-for-age Z-score (WAZ), height-for-age Z-score (HAZ), BMI-for-age Z-score (BMIZ), weight-for-height Z-score (WHZ), head circumference (HC), growth velocities, underweight, stunting, and wasting. Multivariable regression models examined the associations between BLLs and growth outcomes, with adjustment for potential confounders. The geometric mean BLLs was 59.3 μg/L and 82% of children had BLLs >35 μg/L at the age of 12.8 months. After adjusting for confounding factors, no overall association was found between BLL quartiles and HAZ, WAZ, BMIZ, WHZ, growth velocities, wasting, and underweight. However, boys in the highest quartile had a 1.02 cm lower HC (95% CI: [−1.81, −0.24]) as compared to the lowest quartile. Furthermore, an increased odds of being stunted was observed in children in the highest quartile of exposure compared to the first (OR: 2.43; 95% CI: [1.11–5.33]) which remained statistically significant only among girls in sex-specific strata. Blood lead was found to be associated with an increased risk of childhood stunting and a lower head circumference in a resource-limited setting.

## 1. Introduction

Lead poisoning is a major problem that affects children worldwide, particularly in developing countries [1]. Lead exposure has been associated with adverse health effects, including poor neurocognitive development, behavioral problems, and the risk of cardiovascular diseases in adult life [2,3,4,5]. The Centers for Disease Control and Prevention (CDC) have set a reference value of 35 μg/L to identify children with blood lead levels (BLLs) higher than most children’s levels to recommend the initiation of public health actions [6]. Although these reference values are important in identifying children with higher BLLs, there are no identified BLLs considered safe as there is a growing body of evidence that lower BLLs are associated with adverse health outcomes [2,7].

Lead exposure, in 2016, was estimated to account for 63.2% of the global burden of developmental intellectual disability, 10.3% of the global burden of hypertensive heart disease, 5.6% of the global burden of ischemic heart disease, and 6.2% of the global burden of stroke [8,9]. Furthermore, estimates indicate that in 2017, lead exposure was responsible for 1.06 million deaths and 24.4 million years of disability-adjusted life years (DALYs) worldwide due to long-term effects on health [8].

Infancy and childhood are considered the most susceptible period of exposure to lead. Children can be exposed to lead from multiple sources, including occupation, contaminated food, drinking water, and herbal medicinal products [10,11,12,13,14,15]. Ancient sources such as leaded gasoline, which was used globally before it was banned, were one of the significant contributors to lead poisoning. Residual emissions from leaded gasoline remain a source of lead exposure via contamination of soils, dust, and foodstuff. Furthermore, the use of old lead pipes or lead-soldered joints in either the distribution system or individual houses is known to contaminate water [12,16,17,18]. BLLs have been widely used as a biomarker of lead exposure largely because of their feasibility [19]. Therefore, most of the information on human exposure to, and the health effects of, lead are based on BLLs. The blood concentration of Pb remains the most commonly used biomarker in a general medical setting and public health surveillance [20].

Growth effects associated with lead are decreased birth weight and size and decreased anthropometric measures in children [18]. Some epidemiological studies have investigated the adverse effects of lead exposure during early periods of development on child growth. Some studies reported a negative association [21,22,23,24,25] between lead and child growth outcomes while other studies found no associations [26,27,28,29,30]. Existing evidence comes from studies that have been carried out in well-resourced countries, where regulations to control and eliminate lead are in place. In contrast, there is scarce evidence from low- and middle-income countries [31,32], where the prevalence of lead exposure and poor child growth is comparatively high.

Within a birth cohort in south Benin, a high prevalence of BLLs >50 µg/L was observed in 1-year-old infants from 2011-13 [12]. BLLs remained elevated when children were six years old [11]. Given the elevated BLLs and the fact that the effect of postnatal lead exposure on child growth has not been well studied, particularly in low- and middle-income countries, this study attempts to accomplish the following objectives:

1. To investigate associations between BLLs at the age of 12.8 months and the following growth outcomes at ages 4 and 6 years in Beninese children: weight-for-age Z-score (WAZ), height-for-age Z-score (HAZ), BMI-for-age Z-score (BMIZ), weight-for-height Z-score (WHZ), and head circumference (HC) assessed at age 6 years only.
2. To examine associations between childhood BLLs at the age of 12.8 months and underweight, stunting, and wasting at ages 4 and 6 years.
3. To evaluate associations between childhood BLLs at the age of 12.8 months and growth velocities at ages 4 and 6 years.

## 2. Materials and Methods

### 2.1. Study Design and Population

The current analysis was based on the data from the Allada cohort study. This study was carried out in three health centers (Allada, Attogon, and Sekou) in the district of Allada, a semi-rural district located in the south of Benin. Children were born to women enrolled in a randomized clinical trial (Malaria in Pregnancy Preventive Alternative Drugs, MiPPAD, NCT00811421) [33]. The original population comprised 961 children for whom detailed information is provided elsewhere [34]. The biomonitoring data on BLLs at age 1 year were not available for 300 children and were excluded from the analyses; therefore, 661 children with available data on BLLs were included in the present investigation. In addition to height and weight growth data, head circumference measurements were also available at age 6 years for a subset of 370 children, which was included in the present investigation.

### 2.2. Exposure and Data Collection

Blood lead levels were measured in whole blood collected from children aged 1–2 years old. BLLs were analyzed at the Centre de Toxicologie, Institut National de Santé Publique du Québec (INSPQ, Québec, Canada). All BLLs were analyzed by inductively coupled plasma mass spectrometry (ICP-MS) after dilution of blood samples with a detection limit of 0.2 µg/L. The analytical methods are described elsewhere (Bodeau-Livinec et al., 2016).

The parent characteristics studied were economic status (measured by family wealth score), maternal education, estimated preconception body mass index (BMI) [35], gestational age assessed by Ballard score, and gravidity. Information on family wealth at age 1 year was assessed through a checklist of material possessions (such as a car, motorbike, bike, television, cow, and radio), which was later transformed into a wealth scale, with scores ranging from 1 to 15 [12].

The child characteristics assessed were child sex, birth weight, and iron deficiency at 1 year. Iron deficiency was defined as serum ferritin concentrations <12 µg/L in the absence of inflammatory conditions assessed by C-reactive protein (CRP) levels within range (<5 mg/L). Serum ferritin concentrations between 12 and 70 µg/L in the presence of elevated CRP levels (>5 mg/L) were used as a threshold for iron deficiency.

### 2.3. Outcomes

Weight and height were measured between birth and age 6 years in the maternity clinic by trained staff, with children wearing light clothes only and no shoes. Detailed measurement procedures are described elsewhere [34]. Head circumference (HC) was measured with a measuring tape at age 6 years only.

The Jenss–Bayley growth model, which previously presented the best goodness-of-fit on the weight and height of these children [34], allowed prediction of the weight, height, and momentaneous weight and height velocities at the same ages (between 1 and 6 years) for all children. Growth velocity, which is a more dynamic measure of growth, was predicted using the first derivative of the individual equation [36]. For this research, model-predicted height/weight, momentaneous growth velocities at ages 4 and 6 years and observed HC (only at age 6) were used in the analysis. Based on the predictions, the height-for-age Z-score (HAZ), weight-for-age Z-score (WAZ), BMI-for-age Z-score (BMIZ), and weight-for-height Z-score (WHZ) (hereafter termed growth dimensions) of children were calculated, using the updated 2007 WHO Child Growth Standards as a reference. Children with HAZ < −2, WAZ < −2, and WHZ < −2 were categorized as underweight, stunted, and wasted, respectively [37], which are equally used as outcomes of interest when examining associations with BLLs.

### 2.4. Statistical Analysis

Mean values (HAZ, WAZ, BMIZ, WHZ, and HC) are presented with the proportion of children stunted, wasted, and underweight. Child BLLs at age one are described in terms of the geometric mean and proportion of children with BLLs beyond 35 μg/L.

Crude and multivariable regression models evaluated associations between blood lead levels, as the exposure variable, and growth dimensions (HAZ, WAZ, WHZ, and BMIZ) and HC as the continuous outcomes. Analyses were conducted using quartiles of BLLs to account for non-linear associations and repeated using continuous BLLs to study linear trends. Multivariable models were adjusted for potential confounders based on crude associations (data not shown) and a priori variable selection.

Models were adjusted, based on the literature, for maternal characteristics (education, material possession/wealth score, estimated pre-conceptional BMI (kg/m2) and child characteristics (sex, low birth weight, and iron deficiency at age 1) [38,39,40]. Because iron deficiency may increase susceptibility to elevated BLLs [41] and is also known to be associated with food insecurity [42], iron deficiency (yes, no) was additionally adjusted in the final models.

Associations are presented with 95% confidence intervals (CIs). Breastfeeding was not adjusted for in the models as almost all children were breastfed. Multiple linear regressions were run to assess the associations between BLLs and growth outcomes. We included children with complete data on BLLs at one year of age. We tested for effect modification by sex by calculating the *p*-value of the product term between child sex and BLLs.

In additional analyses, multiple logistic regression models were run to assess the independent relationship between BLLs at age 1 and three forms of undernutrition (stunting, wasting, and underweight) both at age 4 and 6 years. Models were further adjusted for child weight (only for stunting) and height (only for underweight) but not both variables in the same model to avoid multi-collinearity [32]. BLLs were compared between children with undernutrition and those without undernutrition using the Wilcoxon rank-sum test.

All statistical analyses were performed using STATA 16.1 (StataCorp. 2019. Stata Statistical Software: Release 16. College Station, TX: StataCorp LP). The statistical significance level was set at *p* < 0.05.

## 3. Results

### 3.1. Study Population Characteristics

The final study sample comprised 661 children, with an almost equal proportion of boys (50.5%) and girls (49.5%). Table 1 describes the demographic and clinical characteristics of the study population. The mean maternal age at childbirth was 25.6 years. In terms of educational level, 85.6% of mothers could not read or write. In total, 16.2% of mothers were underweight before pregnancy. At birth, children weighed on average 3.04 kg (SD, 0.40) with an average length of 49.1 cm (SD, 2.20). Most children (93.1%) were born full-term and almost all of them were exclusively breastfed till 6 months. The proportion of stunted children were 6.1% (*n* = 40), 27.8% (*n* = 182), 27.6% (*n* = 181), and 1.85% (*n* = 12) at age 1, 2, 4, and 6 years, respectively. The proportions of wasted children were 4.1% (*n* = 27), 1.5% (*n* = 10), 5.0% (*n* = 33), and 32.7% (*n* = 211) at age 1, 2, 4, and 6 years, respectively. The proportions of children underweight at age 4 and 6 years were 24.4% (=159) and 16.6% (*n* = 108). Mean WAZ and HAZ in children at the age of 6 years were −1.24 (SD, 0.78) and −0.31 (SD, 0.83), respectively.

The average age for BLLs assessment was 12.8 months (range: 12 to 24 months). The geometric mean BLL at age one was 59.3 μg/L (median, 10th–90th centile: 55.7, 30.5–129) and 82% of children presented with BLLs > 35 μg/L.

### 3.2. Association between BLLs at Age 1 and WAZ, HAZ, BMI-Z, WHZ, and HC at Age 6

No statistically significant inverse association was found between BLLs quartiles and HAZ, WAZ, BMIZ, or WHZ after adjusting for confounding factors (Table 2). Unadjusted associations between log-BLLs (continuous variable) at age one and head circumference (continuous variable) at age 6 suggested a difference by sex (*p* for interaction term, 0.048). However, the *p*-value for the interaction term was 0.12 in the adjusted analyses. The unadjusted association between log-BLLs and head circumference at age 6 was found to be negative in boys (β = −0.43; 95% CI: [−0.84, −0.02]) compared to girls (β = 0.19; [−0.27, 0.64]). In the adjusted analyses when stratified by child sex, boys in the highest quartile (median BLLs = 113 μg/L) had a 1.02 cm lower HC (95% CI: [−1.81, −0.24]) as compared to those in the lowest quartile (median BLLs: 32 μg/L), with a dose–response trend across quartiles (P_trend_ = 0.02).

**Figure 1:**
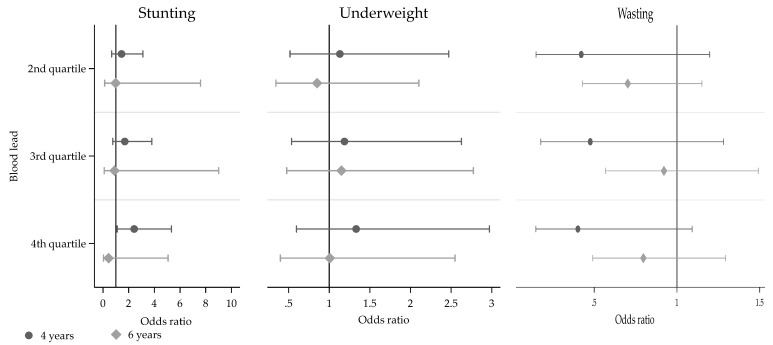
Adjusted odds ratio (OR) ^a^ and 95% confidence intervals for the associations between blood lead level quartiles* at age 1 year and stunting ^a^, wasting ^b^, and underweight ^c^ at age 4 (black shade) and 6 years (grey shade) in Beninese children (Allada cohort). P-_trend_ for stunting ^†^ (4 years: 0.03, 6 years: 0.51). *1st quartile (not shown) is the reference category. ^a^ adjusted for maternal characteristics (education, material possession/wealth score, estimated pre-conceptional BMI) and child characteristics (sex, low birth weight, weight, and iron deficiency at age 1). N for models of 4 and 6 years: 422 and 345, respectively. ^b^ adjusted for maternal characteristics (education, material possession/wealth score, estimated pre-conceptional BMI) and child characteristics (sex, low birth weight, and iron deficiency at age 1). N for models of 4 and 6 years: 553 and 614, respectively. ^c^ adjusted for maternal characteristics (education, material possession/wealth score, estimated pre-conceptional BMI) and child characteristics (sex, low birth weight, height, and iron deficiency at age 1). N for models of 4 and 6 years: 419 and 41, respectively. ^†^ P _trends_ across quartiles of Pb were obtained by inserting its quartile as a continuous variable in the regression model.

**Figure 2:**
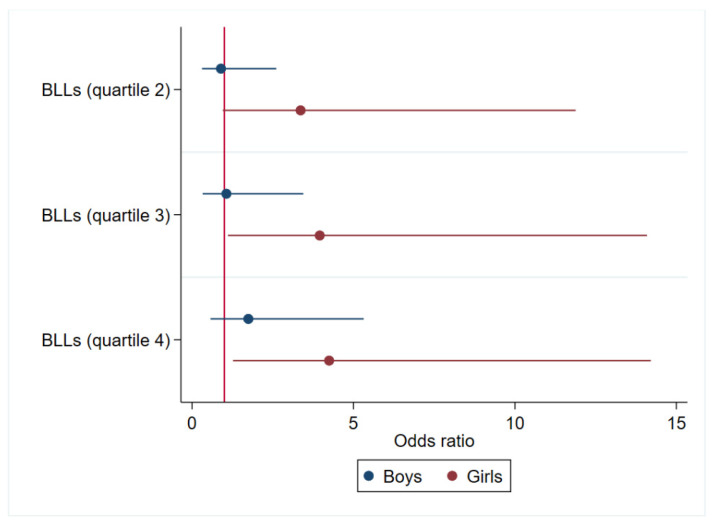
Adjusted odds ratio (OR) ^a^ and 95% confidence intervals for the associations between blood lead level quartiles* at age 1 year and stunting (HAZ < −2) at age 4 years in Beninese children (Allada cohort) stratified by child sex^b^. BLLs: blood lead levels. P_-trend_ ^†^ (boys: 0.27, girls: 0.02). *1st quartile (not shown) is the reference category. ^a^ adjusted for maternal characteristics (education, material possession/wealth score, estimated pre-conceptional BMI) and child characteristics (sex, low birth weight, weight, and iron deficiency at age 1). ^†^ P _trends_ across quartiles of Pb were obtained by inserting its quartile as a continuous variable in the regression model. ^b^ n = 218 for boys; n = 200 for girls.

A statistically significant increased odds of being stunted was observed in children in the highest quartile of exposure as compared to first quartile (unadjusted OR: 1.83; 95% CI [1.11–3.02]) (Supplementary Table S1). This association remained significant after adjusting for potential confounders (Figure 1). Furthermore, a dose–response relationship between BLL quartiles and stunting was observed (P_trend_ = 0.03). BLLs were also statistically different between children with and without stunting at age 4 (*p* = 0.04) but not age 6 (*p* = 0.42). In the stratified analyses by sex, a possible effect modification by sex was observed, with a dose–response trend across quartiles observed only in girls (P_trend_ = 0.02) (Figure 2).

In contrast, no significant association was observed with wasting at any age. BLLs were also not statistically different between children with and without underweight and wasting at any age.

Analyses of height and weight growth velocities showed no significant association between BLLs and velocities. However, a trend was observed across the quartiles of exposure (Table 3).

## 4. Discussion

The cross-sectional prevalence of stunting was low during the first year, peaked at 2–4 years, and recovered thereafter. An earlier study among breastfed children in the Gambia also reported significant increases in stunting around 3 years, with declining prevalence thereafter [43], corroborating the current evidence. Overall, no significant association was observed between BLLs at age one and child growth dimensions (age 4 and 6), HC (age 6), and growth velocities (age 4 and 6). However, when analyses were stratified by child sex, boys in the highest quartile had a 1.02 cm lower HC (95% CI: [−1.81, −0.24]) as compared to those boys in the lowest quartile, with a dose–response trend across quartiles. Furthermore, the analyses of BLLs by its quartiles showed that children who belonged to the higher exposure quartiles had relatively higher odds of being stunted at the age of 4 years as compared to the first quartile of exposure OR 2.24 (95% CI: [1.08–4.66]). This association remained significant and stronger only in girls when analyses were stratified by sex.

Differences in the growth of boys and girls during childhood have been noted in the literature [44]. Furthermore, sex-specific associations have also been observed between childhood lead exposure and growth outcomes in young children. For example, a study conducted in China reported a significant inverse association between cord blood lead levels and head circumference only in male newborns [45]. While in another study conducted in the United States, inverse associations were observed between prenatal lead exposure and birth length and head circumference among female infants [46]. In another study, BLLs were negatively associated with WAZ and HAZ in boys but not in girls in China [47] while a study conducted in Canada observed no significant difference in childhood HAZ, WAZ, and BMI-Z in sex-specific strata [23].

This study expands the limited body of evidence on exposure to lead during the early years of development and child growth and is the first to examine the effect of lead exposure on linear growth in this population in Benin. Previous studies that investigated lead exposure and child growth reported inconsistent findings. A cross-sectional analysis of data from the Third National Health and Nutrition Examination Survey, 1988–1994 reported significant negative associations between blood lead concentration and stature and head circumference among children aged 1 to 7 years [48]. However, findings from the UK observational birth cohort study reported no strong evidence for any associations of prenatal lead exposure with z-scores for BMI, height or weight, and head circumference from age 4 to 61 months [49]. A community-based cross-sectional study [24] conducted among children aged 6–59 months in Kampala, Uganda reported a negative association between concurrent blood lead and HAZ. However, this study was based on a small pilot sample of 100 children and evaluated only one growth dimension (HAZ). In addition, maternal BLLs assessed during pregnancy have been reported to be negatively associated with height for age and weight for age in children aged 4–6 years in Mexico [22]. Another cross-sectional study observed no associations between hair concentrations of lead and the growth dimension of children (*n* = 324) from homeless families aged < 6 years living in shelters for people without housing in France [28]. The population of this cross-sectional study had a higher proportion of children with BMI-Z < 2 standard deviations (90.6%), with more than half of the children suffering from food insecurity [28].

The current study observed a statistically significant association between BLL quartiles and stunting at age four, with a significant exposure–response relationship. A study of 729 slum-dweller children aged < 2 years with high a prevalence of stunting and elevated BLLs, i.e., >50 μg/L (39% and 86.6%, respectively), reported elevated BLLs as a significant predictor of stunting but not wasting [32], which corroborates our findings. One of the proposed mechanisms underlying poor physical growth is impairment of bone growth [18] and disruption of growth hormone secretion [50].

Inconsistencies between the current study results and those from prior studies are likely due to several factors, including differences in the study design and study populations. Previous studies from less developed countries that reported negative associations between Pb exposure and child growth outcomes differed in the prevalence of undernutrition and exposure levels. The exposure levels of Pb and the proportion of children stunted were generally lower in the current study than those reported in prior studies, possible leading to lower power and thus, thresholds may not have been reached for the previously reported growth outcome effects related to Pb in the current study. Indeed, the association between BLLs at age 1 and HAZ at later ages (2 and 4 years, where more children were stunted as compared to age 1 and 6 years) shifted towards modest significance (data not shown). Similarly, the study population in an urban settlement in Uganda had a high prevalence of stunting at 22.7% [24], compared to our semi-rural population in Benin (6%), and a higher prevalence of elevated BLLs at 65% vs. 58.1% in our study.

One of the strengths of this study includes the use of modeled growth data of a long-term prospective cohort, which is one of the few cohorts conducted in sub-Saharan Africa. Furthermore, growth is a complex process; there are several possible indicators used to assess child growth, and we were able to use a variety of them, including stature, weight, BMI, head circumference, growth velocities, and undernutrition status at two different age points when the examining association between lead exposure and child growth. This is very pertinent given that malnutrition is an important public health problem in this setting. We assessed lead exposure around infancy, which is an important period in terms of a toxicological point of view. An important limitation of this thesis is external validity. The data comes from a study that lacked a representative sample. Therefore, the generalizability of the findings is not guaranteed.

## 5. Conclusions

Overall, this study did not find any statistically significant inverse association between lead exposure in infancy and z-scores for BMI, height, or weight at ages 4 and 6 years in the adjusted models. Furthermore, no significant inverse associations were observed between infantile lead exposure and height or weight velocities at ages 4 and 6 years. However, high exposure quartiles were associated with stunting at age 4 years and this association remained significant only in girls in the sex-specific analyses. Finally, high-exposure quartiles were also associated with a lower head circumference in boys only, indicating sex-specific associations.

## Figures and Tables

**Table 1:** Study population characteristics (*n* = 661).

Characteristics	Category	*n* (%) or Mean/Median (SD)
Parental characteristics		
Gravidity		
	primigravida	116 (17.6)
	multigravida	129 (19.5)
	Grand multigravida	416 (62.9)
Maternal Education		
	Primary education or more	94 (14.4)
	No education	560 (85.6)
Maternal age at childbirth (years)	<22 22–25 26–30 >31	173 (26. 2) 178 (26.9) 191 (28.9) 119 (18.0)
Pre-pregnancy BMI (kg/m^2^)		
	Underweight (<18.5)	107 (16.2)
	Normal (18.5–24.9)	481 (72.8)
	Overweight (25–29.9) or obese (> 30)	73 (11.0)
		21 (3.2)
Gestational age (weeks)		
	<37	45 (6.9)
	>37	606 (93.1)
Family wealth score at age 1 (tertiles)		
	Lowest	268 (41.0)
	Medium	195 (29.8)
	Highest	191 (29.2)
Child characteristics		
Sex		
	Male	333 (50.5)
	Female	326 (49.5)
Low birth weight		
	Yes	54 (8.5)
	No	579 (91.5)
Birth length		49.1 (2.2)
Age of child at lead assessment (median, months)		12.0 (1.87)
Low birth weight (<2.5 kg)		
	Yes	54 (8.5)
	No	579 (91.5)
Blood lead level (median, μg/L)		55.7 (61.7)
Exclusive breastfeeding for 6 months		
	Yes	646 (97.7)
	No	12 (1.8)
Anemia at age 1 year Hb < 110 g/L		
	Yes	450 (71.5)
	No	179 (28.5)
Iron deficiency at 1 year (ferritin < 12 μg/L or 12–70 μg/L if CRP > 5 mg/L)		
	Yes	293 (44.33)
	No	368 (55.67)
Malaria at age 1 year		
	Yes	65 (10.4)
	No	561 (89.6)
Weight-for-age Z-score (6 years)		−1.24 (0.78)
Height-for-age Z-score (6 years)		−0.31 (0.83)
BMI-for-age Z-score (6 years)		−1.65 (0.87)
Weight-for-height Z-score		−1.67 (0.80)
Head circumference (6 years) (*n* = 370)		49.93 (1.79)
Medical center location		
	Sekou	399 (60.6)
	Attogon/Allada	259 (39.4)

BMI: body mass index; SD: standard deviation. All values are reported as mean/median (SD) or *n* (percentage).

**Table 2:** Linear regression coefficients (95% confidence intervals) for the associations between blood lead levels at age 1 year and growth at age 4 and 6 years in Beninese children (Allada cohort).

Age (Year)	BLLs Quartile *	WAZ		HAZ		BMI-Z		WHZ		HC	
		unadjusted β coeff. (95% CI)	adjusted β coeff. (95% CI)^a^	unadjusted β coeff. (95% CI)	adjusted β coeff. (95% CI)^b^	unadjusted β coeff. (95% CI)	adjusted β coeff. (95% CI)^c^	unadjusted β coeff. (95% CI)	adjusted β coeff. (95% CI)^d^	unadjusted β coeff. (95% CI)	adjusted β coeff. (95% CI)^e^
		*n* = 659	*n* = 652	*n* = 659	*n* = 626	*n* = 659	*n* = 626	*n* = 659	*n* = 626		
*4*	2nd	−0.01 (−0.16, 0.14)	−0.02 (−0.17, 0.13)	−0.06 (−0.21, 0.10)	−0.04 (−0.19, 0.11)	0.05 (−0.11, 0.21)	0.01 (−0.15, 0.18)	0.02 (−0.14, 0.19)	0.001 (−0.17, 0.17)	-	-
	3rd	−0.07 (−0.22, 0.09)	−0.08 (−0.24, 0.07)	−0.08 (−0.24, 0.07)	−0.07 (−0.23, 0.08)	−0.01 (−0.17, 0.15)	−0.03 (−0.19, 0.13)	−0.03 (−0.19, 0.14)	−0.04 (−0.21, 0.13)	-	-
	4th	−0.17 (−0.33, −0.02)	−0.14 (−0.29, 0.01)	−0.17 (−0.32, −0.01)	−0.11 (0.26, 0.04)	−0.08 (−0.24, 0.08)	−0.05 (−0.21, 0.12)	−0.11 (−0.27, 0.06)	−0.07 (−0.23, 0.10)	-	-
	P-_trend_ ^†^	0.02	0.13	0.03	0.14	0.25	0.48	0.16	0.88		
*6*		*n* = 649	*n* = 620	*n* = 649	*n* = 620	*n* = 649	*n* = 620	*n* = 646	*n* = 614	*n* = 370	*n* = 353
	2nd	0.01 (−0.16, 0.18)	−0.01 (−0.18, 0.16)	−0.07 (−0.25, 0.11)	−0.05 (−0.23, 0.13)	0.08 (−0.11, 0.27)	0.05 (−0.14, 0.23)	0.08 (−0.09, 0.25)	0.05 (−0.13, 0.22)	0.22 (−0.29, 0.72)	0.09 (−0.41, 0.59)
	3rd	−0.07 (−0.23, 0.10)	−0.07 (−0.24, 0.10)	−0.11 (−0.29, 0.07)	−0.10 (−0.28, 0.08)	0.02 (−0.17, 0.21)	−0.004 (−0.19, 0.18)	−0.01 (−0.19, 0.16)	−0.01 (−0.19, 0.17)	0.11 (−0.41, 0.62)	0.12 (−0.39, 0.64)
	4th	−0.16 (−0.33, 0.01)	−0.09 (−0.26, 0.08)	−0.19 (−0.37, −0.01)	−0.13 (−0.32, 0.05)	−0.04 (−0.23, 0.15)	0.001 (−0.19, 0.20)	−0.05 (−0.22, 0.12)	0.0004 (−0.18, 0.18)	−0.37 (−0.90, 0.16)	−0.30 (−0.84, 0.23)
	P-_trend_ ^†^	0.05	0.21	0.04	0.13	0.58	0.89	0.40	0.56	0.16	0.33

BLLs: Blood lead levels. * 1st quartile (not shown) is the reference category. ^a,b,c,d,e^ adjusted for maternal characteristics (education, material possession/wealth score, estimated pre-conceptional BMI) and child characteristics (sex, low birth weight, and iron deficiency at age 1). ^†^ P _trends_ across quartiles of Pb were obtained by inserting its quartile as a continuous variable in the regression model. WAZ: weight-for-age Z-score, HAZ: height-for-age Z-score, BMI-Z: BMI-for-age Z-score, WHZ: weight-for-height Z-score, HC: head circumference.

**Table 3:** Regression coefficients (95% confidence intervals) for the associations between blood lead levels at age 1 year and growth velocities at age 4 and 6 years in Beninese children (Allada cohort).

Age (Year)	BLLs Quartile *	Height Velocities (mm/Day)		Weight Velocities (g/Day)	
		unadjusted β coeff. (95% CI)	adjusted β coeff. (95% CI) ^a^	unadjusted β coeff. (95% CI)	adjusted β coeff. (95% CI) ^a^
		*n* = 659	*n* = 626	*n* = 659	*n* = 626
4	2nd	−0.13 (−0.46, 0.19)	−0.11 (−0.44, 0.22)	0.03 (−0.13, 0.20)	0.004 (−0.16, 0.17)
	3rd	−0.19 (−0.52, 0.14)	−0.20 (−0.53, 0.14)	−0.01 (−0.17, 0.15)	−0.01 (−0.18, 0.15)
	4th	−0.29 (−0.61, 0.04)	−0.19 (−0.52, 0.14)	−0.08 (−0.24, 0.08)	−0.03 (−0.20, 0.13)
	P-_trend_ ^†^	0.09	0.22	0.26	0.67
		*n* = 659	*n* = 626	*n* = 659	*n* = 626
6	2nd	−0.13 (−0.46, 0.19)	−0.11 (−0.44, 0.22)	0.05 (−0.12, 0.21)	0.02 (−0.15, 0.18)
	3rd	−0.19 (−0.52, 0.14)	−0.20 (−0.53, 0.14)	−0.01 (−0.18, 0.16)	−0.01 (−0.18, 0.16)
	4th	−0.29 (−0.61, 0.04)	−0.19 (−0.52, 0.14)	−0.08 (−0.24, 0.09)	−0.03 (−0.19, 0.14)
	P-_trend_ ^†^	0.08	0.22	0.29	0.71

* 1st quartile (not shown) is the reference category. ^a^ adjusted for maternal characteristics (education, material possession/wealth score, estimated pre-conceptional BMI) and child characteristics (sex, low birth weight, and iron deficiency at age 1). ^†^ P-_trend_ across quartiles of Pb were obtained by inserting its quartile as a continuous variable in the regression model.

## Data Availability

The data presented in this study are available on request from the corresponding author.

## Supplementary Materials

The following supporting information can be downloaded at: https://www.mdpi.com/article/10.3390/toxics10100595/s1, Table S1: Odds Ratio (OR) and 95% confidence intervals for the associations between blood lead levels at age 1-year and stunting, wasting and underweight at age 4 and 6 years in Beninese children (Allada cohort)
