# Supplementary material for: Lead Exposure in Infancy and Subsequent Growth in Beninese Children
Source: Toxics. 2022 Oct 8;10(10):595. doi: 10.3390/toxics10100595 (PMC9609716; doi:10.3390/toxics10100595)
Supplement: Supplementary file 1 [file toxics-10-00595-s001.zip › toxics-1886425-supplementary.pdf]

Table S1 Odds Ratio (OR) and 95% confidence intervals for the associations between blood lead levels at age 1-year and stunting and wasting at age4 and 6 years in Beninese children (Allada cohort)

**Age (year)**	**BLL quartile***	**Stunting (HAZ < −2)**		**Wasting (WHZ < −2)**		**Underweight (WAZ < −2)**	
		unadjusted OR	adjusted OR [a]	unadjusted OR	Adjusted OR [b]	unadjusted OR	Adjusted OR [c]
4	2nd	1.52 (0.92–2.53)	1.44 (0.67–3.11)	0.45 (0.17–1.21)	0.42 (0.15–1.20)	1.01 (0.59–1.71)	1.13 (0.52–2.47)
	3rd	1.68 (1.02–2.78)	1.70 (0.76–3.80)	0.53 (0.21–1.37)	0.48 (0.18–1.28)	1.44 (0.87–2.38)	1.19 (0.54–2.63)
	4th	1.83 (1.11–3.02)	2.43 (1.11–5.33)	0.53 (0.21–1.36)	0.40 (0.15–1.09)	1.33 (0.80–2.22)	1.33 (0.60–2.97)
	p-trend †	0.10	0.04	0.06	0.02	0.29	0.48
6	2nd	0.75 (0.17–3.43)	0.98 (0.13–7.59)	0.68 (0.42–1.08)	0.70 (0.43–1.15)	0.88 (0.48–1.61)	0.85 (0.34–2.10)
	3rd	0.75 (0.17–3.43)	0.89 (0.09–9.01)	0.90 (0.57–1.42)	0.92 (0.57–1.49)	1.36 (0.77–2.40)	1.15 (0.48–2.77)
	4th	0.52 (0.09–2.86)	0.44 (0.04–5.07)	0.90 (0.57–1.42)	0.80 (0.49–1.29)	1.05 (0.58–1.91)	1.01 (0.40–2.55)
	p-trend †	0.39	0.20	0.93	0.17	0.91	0.89

* 1st quartile (not shown) is the reference category. [a] adjusted for maternal characteristics (education, material possession/wealth score, estimated pre-conceptional BMI) and child characteristics (sex, low birth weight, weight and iron deficiency at age 1).[b] adjusted for maternal characteristics (education, material possession/wealth score, estimated pre-conceptional BMI) and child characteristics (sex, low birth weight, and iron deficiency at age 1).[c] adjusted for maternal characteristics (education, material possession/wealth score, estimated pre-conceptional BMI) and child characteristics (sex, low birth weight, height, and iron deficiency at age 1).† *P* trends across quartiles of Pb were obtained by inserting natural log-transformed BLL as a continuous variable in the regression model.
